# TMS is needed in Public Mental Health Services

**DOI:** 10.1177/10398562251393795

**Published:** 2025-10-30

**Authors:** Saxby Pridmore, Paul B Fitzgerald, Gregory M Peterson

**Affiliations:** Discipline of Psychiatry, 3925 University of Tasmania, Hobart, TAS, Australia; School of Medicine and Psychology, 2219 Australian National University, Canberra, ACT, Australia; School of Pharmacy and Pharmacology, 3925 University of Tasmania, Hobart, TAS, Australia

**Keywords:** transcranial magnetic stimulation, treatment resistant depression, mental health services

## Abstract

Transcranial magnetic stimulation (TMS) is essential for the comprehensive treatment of major depressive disorder (MDD). Around Australia, it is widely available in private practice, but in few Public Mental Health Services. TMS is a revolutionary treatment. Using electromagnetic apparatus, the function of precise local and distant regions of the brain can be modulated, with minimal side effects. Half of patients with MDD do not respond to antidepressant medication. However, a large proportion of medication non-responders can be brought to remission using TMS. This intervention is cost-effective and availability in public practice should be increased.

Major depressive disorder (MDD) is a painful, serious problem, and about half those treated do not respond to medication. Transcranial magnetic stimulation (TMS) is frequently effective in bringing remission to this group. (Overseas, it is also effective in the treatment of some other disorders for which it is not yet approved in Australia).

TMS has been made available through private facilities around Australia, in all capital cities and some regional cities (Launceston and Mildura). However, it is rarely available in Public Health Services. For example, at the time of submitting this manuscript, TMS was available in Tasmania at 4 private (3 outpatient and 1 inpatient) facilities, and zero Public Health Service facilities. In New South Wales, an occasional large hospital may provide some TMS service; however, the Hornsby, Royal North Shore, Southerland and Nepean Hospitals, all teaching hospitals, have (we have been told) no TMS services.

Depression affects around 280 million people globally, with a lifetime prevalence of MDD of 20%.^1^ Such disorders frequently ruin lives, and 3.5% of those suffering MDD die by suicide. MDD is a major problem not only for afflicted individuals, but the community more broadly, through loss of productivity, and health and parental assistance costs.

Treatment resistant depression (TRD) is a more troublesome form of MDD. It is defined/identified when the patient fails to respond to courses of two different antidepressants, provided at recommended doses for the recommended periods. Not only is the acute phase of TRD more difficult to bring to remission, but the rate of relapse is much greater in this group. About half those with MDD suffer the TRD variety.^2^ The medical costs of those with TRD are about 40% higher than those without TRD because of higher utilization of medical systems.^3^

The first placebo-controlled trial of the TMS treatment of MDD was reported in 1996.^4^ A positive outcome was demonstrated but stronger evidence was required and came with further blinded studies a little more than a decade later.^5,6^ More recently, a study of 5010 ‘real-world’ MDD patients reported response rates of 58%–83% and remission rates of 28%–62%.^7^

The Australian Therapeutic Goods Administration (TGA) approved the use of TMS in the treatment of patients with TRD in 2007. The USA Food and Drug Administration (FDA) gave TMS approval in 2008. The FDA has also approved TMS for the treatment of OCD and smoking cessation, and research suggests it may have a role in the treatment of PTSD and auditory hallucinations. The TGA has only approved TMS for TRD, but other applications are expected to be approved. Some authorities have called for TMS to be made available as a first-line treatment of MDD.^8^ These potential changes increase the urgency for TMS skilled clinicians and equipment to be available in the Public Health Systems.

TMS uses electromagnetic theory and equipment to deliver small magnitude electrical activity to precise regions of brain cortex, which communicate with various distant sites. Moderation of the function of local and distant synapses and axonal connectivity underpin clinical improvement. Active TMS increases the expression of genes SCN1A, SNAP25 and PVALB, which are believed to underpin neurophysiological change and clinical recovery.^9^

The advantages over ECT include that memory problems and seizure do not occur. The services of an anaesthetist are not required. To this point, TMS has received little attention in some medical schools and many specialist training centres. This is expected to change with the recent release of consensus and authoritative teaching documents.^10–12^

The fact TMS is not currently available at most Australian Public Mental Health Services may be due in part (the authors speculate) to a lack of familiarity with the treatment among influential senior clinicians, and a mistaken belief of low cost-effectiveness. Financial considerations may also have been important from the private practice perspective, in that TMS could be construed as a marketable service.

Fostering the belief that TMS is prohibitively expensive is that TMS devices require upfront investment and an operator/technician must be on-hand throughout each treatment. However, TMS has been found cost-effective compared to a range of other forms of the management of TRD associated with both unipolar (MDD) and bipolar disorder (BD).^13,14^ A recent report compared the costs of TMS and non-TMS treated cohorts.^15^ While the TMS cohort incurred more hospital outpatient visits, with higher outpatient costs, this group had many fewer inpatient admissions and emergency room visits and returned health and economic dividends through less high acuity hospital visits. In addition, newer forms of TMS involving the patterned application of TMS pulses such as ‘theta-burst stimulation’ have been shown to produce the same therapeutic benefits in markedly shorter periods of time (potentially less costly).

In summary, MDD is a painful disorder which may lead to suicide. About half those suffering this disorder have a treatment resistant form (TRD) which does not respond to standard antidepressant medication and at present, typically requires individuals to undergo expensive ECT, with its common and concerning array of side effects.

TMS is a unique treatment of mental disorders – a method of electromagnetically impacting precise regions of the brain with ‘no metabolic and systemic side effects’.^15^ It frequently provides relief when patients have failed to respond to medication.^4–7,10^ TMS is relatively new to the field (albeit approved in Australia for the treatment of TRD in 2007) and employs technology relatively unfamiliar to psychiatry. It has been assumed TMS is more expensive (or less cost-effective) than other forms of treatment – but this is incorrect.^13–15^

TMS is available around Australia in private practice. To this point, however, few Public Mental Health facilities can provide this service to seriously ill patients, including those without the means to access private treatment, erecting significant financial barriers to equitable care. There are obstacles to be overcome – staff need a few hours of instruction and exposure to apparatus, and apparatus needs to be acquired. These are not insurmountable issues – we encourage psychiatrists, other mental health workers, patients and patient advocates, to find a path enabling the provision of this important service to an unfortunate, rapidly growing number of Australians. This may involve speaking with some politicians and appropriate ‘influencers’.
